# Cultural roots of the myopia boom in Confucian Asia and their implications

**DOI:** 10.1057/s41271-024-00513-1

**Published:** 2024-08-10

**Authors:** Fabian Yii

**Affiliations:** 1 Centre for Clinical Brain Sciences, The University of Edinburgh, Edinburgh, UK; 2 Robert O Curle Ophthalmology Suite, Institute for Regeneration and Repair, The University of Edinburgh, Edinburgh, UK

**Keywords:** Myopia, Prevention, Education, Policy, East Asia, Cultural influence, Confucian values

## Abstract

Current evidence implicates educational pressures and reduced outdoor time as major causes of myopia. This paper examines the ongoing battle against the myopia epidemic in East Asia, including its cultural offshoots such as Singapore, where over 80% of young adults are myopic. East Asian societies share deeply rooted Confucian values that attach great importance to education and familial obligations, with heavy parental investment in education and the perception that academic excellence reflects filial piety. Coupled with a strong emphasis on standardised test results, East Asian children face intense educational pressures from a young age. Existing education-based myopia prevention strategies focus either on top-down school reforms to promote more outdoor time for students during school hours or on bottom-up awareness initiatives encouraging lifestyle changes. However, the entrenched Confucian worldview suggests that more extensive top-down reforms aimed at reducing competition in education, combined with widespread bottom-up awareness initiatives targeting the public—particularly parents, given their active involvement in children’s education outside of school—may be required to truly turn the tide on myopia.

## Key messages

- Asian societies in which myopia prevalence has reached epidemic proportions share deeply rooted Confucian values that place great importance on education and familial obligations.
- More extensive top-down reforms focusing on reducing competition in education—perhaps by reducing the emphasis on high-stakes college/university entrance exams—coupled with widespread bottom-up awareness initiatives targeting the public, particularly parents, are required to better mitigate the myopia epidemic in East Asia.
- The parallels in social attitudes towards education across Confucian societies suggest that Vietnam may similarly experience an explosion of school myopia following its relatively recent expansion of mass education.

## Introduction

**Fig. 1:**
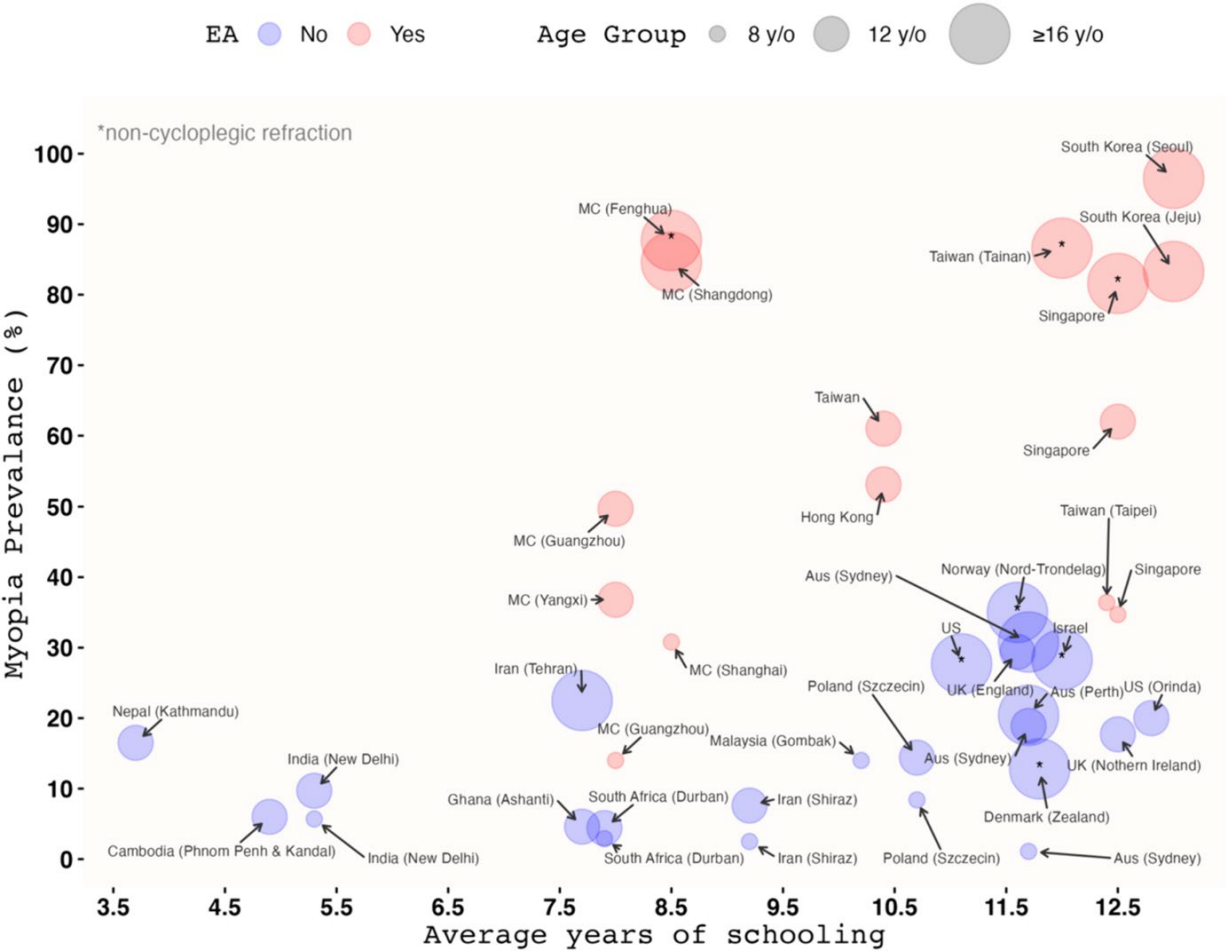
Myopia prevalence in different age groups and regions, based on data from studies reviewed by Matsumura and colleagues [7], against the average years of formal education (schooling) of a given population at the time of data collection. Data pertaining to the average years of schooling are derived from OurWorldInData.org [8]. For the ≥ 16 age group, the upper age limit varies across studies, with a minimum of 17 and a maximum of 29 (mean age ranges from 17 to 22*.*5). Note that for the 8-year-old and 12-year-old age groups, only data from studies using cycloplegic refraction were included, as non-cycloplegic refractive error measurements could overestimate myopia prevalence in younger children by around 20% [9]. None of the existing Japanese studies measured refractive error in children under cycloplegia, so data from Japan are not depicted. However, a recent study [10] reported a very high prevalence of myopia (non-cycloplegic autorefraction) among 8-year-old (78*.*6%) and 12-year-old (94*.*6%) Japanese schoolchildren. Assuming an overestimation of 20% due to the lack of cycloplegia, these estimates still translate to a very high prevalence of 58*.*6% and 74*.*6%, respectively. EA, East Asia and Singapore; MC, mainland China; Aus, Australia; UK, United Kingdom; US, United States; y/o, years old

The precipitous rise in the global prevalence of myopia, projected to reach 50% by 2050, has been aptly described as the “myopia boom” [1]. East Asian regions, including mainland China, Hong Kong, Taiwan, South Korea and Japan, as well as Singapore in Southeast Asia (hereafter collectively referred to as East Asia), are disproportionately affected, with studies estimating myopia prevalence to be 80% or higher among young adults in these regions (Fig. 1). Far from being a benign vision problem, myopia has significant public health implications due to its association with myriad visually debilitating complications, notably pathologic myopia, which is a leading cause of irreversible blindness in parts of the world [2]. In addition to its deleterious impact on quality of life, myopia has been estimated to cost hundreds of billions of dollars globally each year due to direct health expenditures and lost productivity [3]. Given the absence of evidence for a safe level of myopia [4]—and the fact that myopia severity is currently the sole modifiable risk factor for pathologic myopia during childhood [5]—measures aimed at preventing myopia-related complications should focus on preventing or delaying myopia development from the outset [6].

## Education is a major cause of myopia

Myopia prevalence or severity increases with more years spent in education and among students from more academically orientated classes or schools [11]. This has long led to the belief that educational pressures cause myopia, a hypothesis subsequently confirmed by Mendelian randomisation showing that children with a genetic predisposition to more schooling (being more studious) are more likely to be myopic, but not the other way around [12]. The prevailing model of education in East Asia is characterised by high levels of academic pressure, potentially contributing to the myopia epidemic in this region [13].

Although the exact underlying mechanisms remain to be elucidated, such as whether near work plays a mediating role [14], recent research suggests that the causal effect of education on myopia can be partly (40%) explained by reduced outdoor time [15]. Indeed, several school-based cluster randomised trials have confirmed the efficacy of *increased* outdoor time in lowering myopia incidence [16–18]. This protective effect is likely due to the increased release of retinal dopamine in brighter outdoor conditions [18], as light-induced dopamine has been shown to be protective against myopia in various animal studies [19]. Other aspects of outdoor environments may also play a role. For example, outdoor visual scenes tend to create more myopic defocus patterns (images form before rather than after the retina, acting as a “stop” signal for eye growth) that vary little with eye movements and thus are less favourable for myopia development [4].

## Current education-based approaches to myopia prevention

Taiwan, Singapore and mainland China have implemented various school reforms or education-based policy interventions to alleviate educational pressures and promote more outdoor time, aiming, among other things, to lower myopia incidence in schoolchildren. In Taiwan, the Tian-Tian 120 (天天120 or everyday 120) programme introduced by the Ministry of Education in 2010 encourages 120 min of outdoor time daily during school hours, a policy subsequently found to be followed by a *reversal* of the long-term trend towards increasing prevalence of myopia (using prevalence of poor uncorrected vision as a surrogate for myopia prevalence) [20]. Unlike the top-down approach taken in Taiwan, Singapore employs a more bottom-up strategy focusing on public education about the importance of more outdoor time for schoolchildren and the risks of sight-threatening complications associated with myopia through its National Myopia Prevention Programme, which has helped lower the prevalence of childhood myopia to some extent [21].

Since 2018, mainland China has also announced several top-down school reforms, calling for 1 to 2 h of outdoor time daily for schoolchildren and a reduced burden of schoolwork (e.g. no written homework for students in the lower forms), with clearly laid out targets of reducing myopia prevalence by 0.5% and 1% annually over the next 5 years in provinces with low and high baseline prevalence of myopia, respectively [9]. This was soon followed by the “Double Reductions” policy in 2021, which outlawed for-profit after-school tutoring and limited the operating hours of the not-for-profit tutoring sector [9]. In November 2023, Xi’an and Shenzhen became the latest cities in mainland China to mandate that schools incorporate 1 h of physical education daily and no less than 30 min of break between long classes, effective January 2024, with Xi’an’s education bureau emphasising the need for activities outside the classroom “in the sunshine” [22, 23]. South Korea and Japan, on the other hand, have had relatively limited official policy interventions aimed at preventing or delaying myopia onset among schoolchildren, although analogous reforms to reduce school hours (Japan) and curtail the private tutoring sector (South Korea) have been attempted in response to concerns over student health, behaviour and educational equity [9].

## Centrality of education and familial obligations in Confucian Asia

Understanding the historical and cultural context from which the high-pressure East Asian model of education arises is useful for drawing implications for the myopia epidemic and mitigation policies. Notwithstanding contemporary socio-political differences, the lens through which East Asian societies view education is ultimately coloured by their deep-rooted connection to Confucianism [24]—a system of thought propagated by Confucius in the 6th–5th BCE and institutionalised by Imperial China, Japan, Korea and Vietnam to guide various aspects of society and politics for hundreds to thousands of years [25, 26].

Confucianism traditionally placed great importance on education and family, two intertwined elements central to the present discourse. Education was highly valued both abstractly—as a means of self-cultivation through the acquisition of knowledge on proper social etiquette to help maintain social order and—in a more practical sense, as an official avenue (elaborated in subsequent paragraphs) through which ordinary individuals could attain higher social standing [27]. The centrality of family to one’s life is reflected in the expectation that individuals should guide their behaviour in every respect so as to bring honour to or sustain the reputation of their family lineages [28]. This implies that academic success—or failure—of an individual reflects on their entire family and ancestors, providing significant motivation for families to invest heavily in their children’s education, with correspondingly high parental expectations and pressures for children to excel academically [27].

Particularly in imperial China, Korea and Vietnam, a highly complex civil service examination system, or the imperial examination, was implemented as early as over a millennium ago to select the best bureaucrats steeped in Confucian ideals to govern the states [25]. In imperial China, this system was modified from around the seventh century to allow virtually all males to apply for the exams without official endorsement and further modified in the tenth century to eliminate the influence of family or personal connections [25]. These changes gave birth to the world’s first standardised examination system that prioritised merit over birth [29], an important development that opened the door for dramatic upward social mobility for a much wider segment of society than that seen elsewhere in the world [26]. The imperial exams had multiple levels (with a passing rate of less than 1% at the highest level), requiring decades of total dedication from scholars and active (e.g. financial) support from their families [25]. For sons, it was considered an important filial duty to pass the exams [30].

Preparation for the exams began early in life. Children younger than seven embarked on an arduous and highly regimented journey of memorising classical Confucian texts under the auspices of their families, continuing year after year until they could recite them flawlessly by age 15 [25]. This was followed by yet another phase devoted to taking the exams, level by level, with many candidates repeatedly failing and retaking the higher-level exams, so much so that the average age of those attaining the highest degree was around 35 years [25]. The system endured for over (or nearly) a thousand years until as recently as the early twentieth century [25, 26]. Vestiges of the civil exam institution can still be found today. For example, in the Republic of China (Taiwan), the tripartite system of government (legislature, executive and judiciary branches) was adapted to accommodate a fourth branch known as the Examination Yuan, which is responsible for civil service recruitment through state-run exams [31].

Evolving from this unique historical backdrop is the prevailing East Asian worldview that places a disproportionately high premium on education, with high parental academic involvement (parents’ obligation towards children) and expectations—as well as children’s perception that high academic achievement signifies filial piety (children’s obligation towards parents)—giving rise to early-onset educational pressures [30, 32–35]. This worldview extends across social classes, as education was for a long time the single most important path for the “masses” to attain or maintain higher socio-political status. Another important legacy is the development of hyper-competitive, high-stakes college/university entrance examination systems that hark back to the long tradition of the imperial examination [36]. These systems rely solely—or almost entirely—on exam results to determine the tier of tertiary institution a school leaver can apply to, with elite research intensive universities sitting at the top and vocational institutions at the bottom, adding a second source of academic pressure for students [36]. Using mainland China’s *Gaokao* and South Korea’s *Suneung* as examples, cut-off scores to determine eligibility to apply to different tiers of institutions are set each year based on the distribution of scores in that year, and thus the exams may feel like a zero-sum game. The perceived significance of these exams cannot be overstated. For instance, traffic is redirected and construction work is halted near designated test centres, while businesses and stock trading delay openings to minimise disruptions on exam days [37, 38]. The high-stakes nature of these exams is also encapsulated in a famous Chinese saying, “one exam dictates one’s life chances” (一考定终身), which partly explains why some East Asian students prefer to pursue their higher education outside their home countries [27].

Driven by the emphasis on standardised test results and heavy parental investment in education, many East Asian students supplement formal schooling with shadow education, adding a third source of academic pressure [13]. In South Korea, for example, annual spending on private tutoring alone, as a percentage of Gross Domestic Product (GDP), has been reported to be roughly the same as the average percentage spent on tertiary education by members of the Organisation for Economic Co-operation and Development, which primarily comprises wealthy economies [36]. Perhaps not surprisingly, East Asian students consistently top rankings in international standardised tests, such as the triennial Programme for International Student Assessment (PISA) [24]. The Confucian conviction about the central place of education is so ingrained that, as another famous saying goes, families are not prepared to skimp on their children’s education no matter how impoverished they are (再穷也不能穷教育). Combining these deeply entrenched Confucian characteristics with the advent of modern mass education in the twentieth century, it is not surprising that East Asia was among the earliest, if not the first, to experience a boom in myopia prevalence in the ensuing decades.

## Confucian values outside its core cultural sphere

Despite being physically far removed from the core of the Confucian cultural sphere, East Asian diasporas in the West are also known to cling to the same myopiagenic outlook on education, demonstrating the entrenched nature of such cultural traits [39]. While positive hyper-selectivity—or the idea that contemporary immigration selectively attracts Asians who are more highly educated with a success-orientated frame compared to both their non-migrant counterparts and the general population of their host country—may be one reason why East Asian Diasporas tend to espouse myopiagenic values conducive to educational success (the structural explanation) [40], this view has been disputed by some authors who find evidence or arguments in favour of the cultural explanation [41–43].

American students of East Asian origin, known to have higher educational performance, are significantly more likely to engage in shadow education for enrichment purposes—not just to keep up with but to *outperform* their peers—compared to other Asian, White, African and Hispanic Americans [44]. East Asian parents in the United States also demonstrate a stronger commitment to their children’s education based on other measures of investment, such as the availability of home computer for educational purposes, savings for tertiary education and help with homework, even after controlling for family income and other socioeconomic confounding factors [45]. Consequently, these children perceive higher academic expectations and pressure to excel academically in return for their parents’ high investment in their education [45, 46].

Reflecting this higher level of academic pressure, Australian children of East Asian origin aged 6 to 7 years, known to have much higher myopia prevalence, were found to spend less time outdoors (14 h a week) than their White counterparts (21 h a week) in the Sydney Myopia Study [47]. Singaporean Chinese children (3 h per week) spend even less time outdoors—a difference thought to be attributable to higher early-onset academic pressure—coinciding with a significantly higher myopia prevalence compared to Australians of East Asian or White origin [47]. In Malaysia, where the three main ethnic groups usually attend different types of public primary schools catering for their respective mother tongues, children of Chinese descent have a much higher prevalence of myopia (46.4%) than those of Malay (15.4%) or Indian (16.2%) descent [48].

## Implications for education-based approaches to myopia prevention

Viewing East Asian societies and their diasporas through the lens of their deep-rooted connection to Confucian values provides several important implications for myopia prevention strategies. First, given the perceived centrality of education and familial obligations in these societies, a largely *standalone* top-down approach to school reforms may be less effective in mitigating myopia than one *complemented* (not replaced) with widespread bottom-up awareness initiatives targeting the public, particularly parents and possibly schoolteachers. Both groups are highly revered in the Confucian social structure and treated with considerable deference by students, with parents typically being highly involved in their children’s education outside of school. As such, they are important agents of change.

From the perspective of a Confucian society, the balance between the importance of studying and concerns over myopia may be tipped in favour of the former unless there are strong, consistent and concerted efforts directed towards educating the public on the following points: (1) risks of *irreversible,* sight-threatening diseases associated with myopia [2]; (2) absence of a safe level of myopia [4]; (3) critical period during childhood and adolescence when eye growth is active [49], and therefore the risks of myopia and its pathological sequelae may still be amenable [19]; and (4) strong evidence of the protective effect of increased outdoor time against myopia [19]. Without such strong *complementary* efforts, existing top-down approaches, such as tighter regulations on shadow education and increased outdoor time during school hours, could lead to some children compensating for the “lost time” by spending more time studying at home, likely at their parents’ behest, or teachers not complying with official guidelines. Indeed, early top-down reforms in mainland China aimed at reducing the amount of homework and school hours proved ineffective, as schools found ways to circumvent the regulations and parents filled children’s additional free time with private tuition [33].

While existing top-down school reforms appear to be moving in the right direction by encouraging more outdoor time for schoolchildren, additional changes and more extensive adjustments focusing on *reducing competition* in education may be required to reap the full benefits of these school-based efforts. This is particularly important because efforts to encourage more time outdoors in a highly competitive environment that incentivises long hours of studying to excel in high-stakes exams could be seen by children and parents as a conundrum between two evils: risk of myopia versus risk of poorer exam results relative to other students. Therefore, the challenge is to reform the education or examination system so that spending more time outdoors is not perceived as detrimental to school performance and, by extension, life chances—perhaps by rethinking how “performance” is defined and evaluated.

One example is to take a more holistic approach to educational assessment, reducing the emphasis on standardised test results for college/university admissions, something that Japan, South Korea and Taiwan have been considering in recent years [50]. In balancing a less myopiagenic, lower-pressure education system with good educational performance as indicated by PISA rankings, Finland and the Netherlands appear to be reasonable role models [51]. However, determining which specific aspects of the East Asian model of education can be reformed without being overly unpalatable—while ensuring the desired educational outcomes relevant to local or national needs can still be met—is a significant undertaking that requires an interdisciplinary partnership between public health, public policy and social sciences, along with active engagement with stakeholders such as parents.

## Implications for Vietnam

Excluding North Korea due to the lack of myopia prevalence data, Vietnam is the only place in the Confucian cultural sphere that has not experienced a myopia boom of epidemic proportions as seen elsewhere in East Asia and Singapore. That said, it is important to note that mass education in Vietnam took a tumultuous turn during and after the relatively recent long war that wreaked the country in the latter part of the twentieth century (1955–1975), during which it witnessed a considerable decline in school enrolment, particularly from the 1980s to 1990s [52]. However, enrolment rates at all levels, especially among secondary schoolchildren aged 11 to 18 years, have since picked up, increasing from 68.9% (lower secondary) and 16.0% (upper secondary) in 2000 to 97.7 and 59.0% in 2018 [8].

Like others in the Confucian cultural sphere, Vietnamese society attaches great importance to education, seeing it as a mark of familial prestige, along with high parental academic involvement and expectations for children [53]. If not seen in the light of its long historical association with these Confucian values, Vietnam’s relatively low per capita wealth makes it a perplexing anomaly in the international educational arena dominated by other significantly more well-off, resource-rich economies [54]. As a lower middle-income country and the poorest participant in PISA, its historical PISA results, particularly its 2018 rankings for Science (4th), Reading (13th) and Mathematics (24th), were truly impressive [54].

The mainland Chinese experience with myopia may be of relevance for Vietnam. Not too long ago, both places experienced large-scale disruptive events, such as the great leap forward and cultural revolution in mainland China and the Vietnam war in Vietnam. These events were followed by sweeping reforms that helped usher in new eras of relative political and economic stability, with mainland China’s “reform and opening-up” (改革开放) taking the lead in the late 70’s before Vietnam’s “innovation” (*Doi Moi*) about 10 years later. The period of economic reform in mainland China saw a massive expansion of secondary and higher education [47, 55], which was followed by a high prevalence of myopia among semi-rural (46.3%) [56] and urban (73.1%) [57] children aged 15 years from around 2000. Note that mainland China’s GDP per capita at purchasing power parity was only around $3,452 then—noticeably lower than the average of $5,288 in middle-income economies, $34,590 in the European Union and $49,316 in North America [58]. This demonstrates that a massive explosion of school myopia is not predicated on a rich economy but is a problem that Confucian societies are particularly (though certainly not exclusively) vulnerable to following a rapid expansion of mass education. Indeed, wealthy Scandinavian countries like Sweden and Norway are largely spared from the myopia boom [59, 60] despite their relatively early establishment of compulsory schooling, which began as early as the 1920s [61].

Whether Vietnam will follow a similar trajectory a few decades into its *Doi Moi* reform and increasing school enrolment remains an open yet important question. It seems likely that in the absence of effective education-based prevention strategies, Vietnam is on track to become yet another “highly myopic” nation in Asia. Since as early as 2011, the prevalence of myopia among Vietnamese secondary schoolchildren has been around 26.6% (aged 12–15 years) in urban schools and 14.2% (aged 12–16 years) to 16.3% (aged 12–15 years) in rural schools [62, 63], higher than that found in more developed economies with a longer history of full-fledged mass education [7], such as Australia (8.6% and 17.7% in European Australian schoolchildren aged 12 and 17 years from the Sydney Adolescent Vascular and Eye Study between 2009 and 2011) [64].

## Conclusions

Is it a coincidence that societies with the highest prevalence of myopia are all found within the East Asian cultural sphere or their diasporas, including Singapore with its majority ethnic-Chinese population? Perhaps not, if one considers how the centrality of education and familial obligations in the Confucian worldview shared by these societies—where families invest heavily in their children's education and academic excellence is seen as a mark of filial piety—influences the lifestyle of schoolchildren.

While existing education reforms are moving in the right direction by encouraging more outdoor time for students during school hours, more extensive top-down reforms focusing on *reducing competition* in education, perhaps by lessening the emphasis on one-chance college/university admissions exams—coupled with widespread bottom-up *awareness initiatives* targeting the public, particularly parents due to their high level of involvement in children’s education outside of school—may be required to truly turn the tide on the myopia epidemic. The parallels in social attitudes towards education across Confucian societies also suggest that Vietnam, following its relatively recent expansion of mass education, may also experience an explosion of school myopia, underscoring the importance of effective education-based prevention strategies in the country while the trend can still be reversed.

## Data Availability

Not applicable.
